# Depression and quality of life in adults perceiving exposure to parental alienation behaviors

**DOI:** 10.1186/s12955-019-1080-6

**Published:** 2019-01-14

**Authors:** M. C. Verrocchio, D. Marchetti, D. Carrozzino, A. Compare, M. Fulcheri

**Affiliations:** 1 Department of Psychological, Health, and Territorial Sciences, “G. d’Annunzio” University of Chieti-Pescara, Via dei Vestini 31, 66100 Chieti, Italy; 2 Psychiatric Research Unit, Psychiatric Centre North Zealand, Copenhagen University Hospital, Hillerød, Denmark; 3 Department of Human & Social Sciences, University of Bergamo, Bergamo, Italy

**Keywords:** Parental alienation, Adverse outcomes, Health-related quality of life, Depression

## Abstract

**Background:**

The current study is aimed at examining the relationship between exposure to parental alienation (PA) behaviors, depression, and health-related quality of life (HRQoL) in Italian adults.

**Methods:**

Four hundred ninety-one adults were tested. Participants filled out the following self-rating scales: The Baker Strategy Questionnaire (BSQ), the Beck Depression Inventory – II (BDI-II) and its brief version (6-item version of the BDI-II), the Short-Form 36 (SF-36) Health Survey for measuring HRQoL and its brief version including 3 items (WHO-3) of the 5-item World Health Organization Well-Being Index.

**Results:**

Findings revealed statistically significant differences between participants who reported PA and those who did not. Participants who reported exposure to PA behaviors had higher scores on the original BDI-II and its 6-item version (*p* < 0.05, *p* < 0.01, respectively); they had also lower levels of HRQoL as resulting from 6 of the 8 SF-36 domains (at least *p* < 0.05), including lower scores on the WHO-3 (*p* < 0.01). Perceiving an exposure to PA behaviors significantly increased the likelihood of being above the clinical cut-off on the BDI-II (*p* < 0.01), the 6-item version of the BDI-II (*p* < 0.05), and the WHO-3 (*p* < 0.05). Moreover, perceiving an exposure to PA increased the odds of diminished HRQoL (*OR* = 2.43 and *OR* = 1.92 for general health and social functioning domains, respectively).

**Conclusions:**

Childhood exposure to PA was related to higher likelihood of depressive symptoms and diminished HRQoL in adulthood. Our findings suggest the need for preventive and clinical interventions to protect vulnerable children involved in PA from negative outcomes.

## Background

Parental Alienation (PA) is the term used to describe a family dynamic characterized by specific behaviors engaged by one parent (the alienating**/**preferred parent) which could result in a child’s unjustified rejection of the other parent (the targeted parent) [1]. Previous studies identified specific PA behaviors of one parent to turn the child against the other parent and include, among other things, denigrating the other parent, limiting the child’s contact with the other parent, and interfering with communication between the child and the other parent. These behaviors are likely to create a loyalty conflict in the child [1, 2] who may feel pressure to ally himself or herself strongly with the preferred parent and rejects the relationship with the targeted parent without legitimate justification showing dislike, distrust and fear towards him or her [3, 4].

As Bernet and colleagues [5] wrote, “Parental alienation affects hundreds of thousands of children in the United States and comparable numbers around the world. Parental alienation has been recognized by thousands of mental health and legal professionals. It is treated by thousands of psychologists, psychiatrists, social workers, and family counselors. There is no doubt that parental alienation is recognized by the vast majority of mental health professionals who work with children of divorced parents” (p. 142). Although cross-cultural studies show that many aspects of parenting are shaped by cultural factors [6], there is substantial agreement in the literature about the behavioral strategies parents can use to manipulate their children in ways that may interfere with their relationship with the other parent [7].

A large body of literature highlighted both short- and long-term negative consequences of exposure to ABs on mental health, and depression is one of the most widely studied [1, 7–12]. Depression was extensively analyzed as an outcome of PA, but except for one study [13] it was assessed with continuous scores on the BDI-II. That is, only one study was conducted to assess whether an above or below clinical cut-off score on the BDI-II was associated with exposure to PA. The BDI-II cut-off score is a well-established measure of depression, often used to distinguish between normal and clinical range among general population samples. However, Bech et al. [14] showed that the 21-item version of the BDI-II included a mixture of somatic and psychological symptoms of clinical depression without discriminating the severity of such symptoms. Accordingly, the current literature suggests that depression is a heterogeneous clinical condition [15] and collapsing individuals with different symptoms of depression into one undifferentiated category (depressed vs. not depressed) discards much information about the specific nature of symptoms [16].

The negative impact of PA on children, with outcomes ranging from psychopathology (e.g., depression, anxiety, substance abuse, and conduct disorders), to declines in academic performance and low self-esteem are factors that may impair the quality of life (QoL).

QoL is defined as the total subjective perception based on an assessment of the individual’s own life, emotion and cognition processes, and thus it is an expression of an individual’s well-being [17]. From a prevention perspective, health is not only the presence or absence of disease, but rather comprises an individual’s perception of health, functioning, and well-being [17]. QoL has been extensively investigated in medical and psychological research since the 1990s because it is a subjective indicator of health status that goes beyond a diagnosis of mental illness. Several studies [18–21] have shown the consequences of an impaired health-related quality of life (HRQoL) in terms of economic burden in different patient populations (e.g., people with depression, anxiety, bipolar disorder, diabetes, irritable bowel syndrome, etc.). Poor HRQoL is significantly associated with higher health-care costs which are mainly related to increased health care utilization by individuals [21].

In a recent review, Weber and colleagues [22] highlighted that over the past 15 years there has been growing interest in HRQoL research among individuals who have suffered childhood maltreatment and some studies have shown a significant adverse effect of child psychological maltreatment on current HRQoL. PA was conceptualized as a form of psychological maltreatment (PM) [23, 24]. The Diagnostic and Statistical Manual of Mental Disorders [25] defines child abuse as “non-accidental verbal or symbolic acts by a child’s parent or caregiver that result, or have a reasonable potential to result, in significant psychological harm to the child” (p. 719). As Baker and Ben Ami [10] note, “The psychological foundation of parental alienation - lack of empathy and the inability to tolerate the child’s separate needs and perception - is also the foundation of psychological maltreatment” (p. 473). The behaviors of the parent engaging in the PA aligned with the subtypes of PM (spurning, terrorizing, isolating, corrupting/exploiting, denying emotional responsiveness) as defined by the American Professional Society on the Abuse of Children (APSAC) [26]. Moreover, the exposure to these behaviors is likely to result in children feeling worthless, flawed, unloved, unwanted, endangered, or only of value in meeting another’s needs.

Research has linked PA to PM and to negative psychological outcomes in a number of independent samples [8, 10, 27–29]. A recent study [30] extended this body of work by demonstrating that reported exposure to ABs was associated with anxiety both directly and indirectly mediated through psychological maltreatment. To date, no study has evaluated HRQoL or other health outcomes among adults who reported exposure to PA during childhood. Lack of research in this field may be because the impact of exposure to PA behaviors is an area of recent clinical interest. Although the association between PA and PM and its negative outcomes in adulthood has been well established [31], the association between PA and HRQoL facets is still unknown. As mentioned above, research demonstrated a significant adverse effect of child psychological maltreatment on current HRQoL. Since individuals who have suffered from exposure to PA must cope with long-lasting consequences that can affect their daily lives, it seems plausible that PA may be associated with diminished HRQoL as well. Demonstrating this relationship could be used to design more effective preventive and therapeutic interventions targeted for individuals experiencing PA. In this research, we analyzed the association between depressive symptoms, HRQoL, and exposure to PA perceived by Italian adults. Specifically, we addressed the following questions:

1. Is exposure to PA behaviors associated with depressive symptoms and HRQoL rates in a sample of Italian adults from the general population?
2. Is exposure to PA behaviors associated with an increased likelihood of being above clinical cut-off scores for measures of depressive symptoms and HRQoL dimensions?

## Methods

### Participants and procedure

Between October of 2015 and April of 2016, flyers were delivered to a variety of employment, recreational, and university settings in the southern region of Italy as well as distributed to friends and colleagues who were encouraged to forward it to others. The flyers stated that a researcher in the Clinical Psychology Laboratory at University of Chieti (Italy) was “*Seeking anyone over the age of 18 to take part in a study on the relationships between quality of life and parental relationships*”. Interested individuals were invited to contact the researcher via telephone or e-mail. Once individuals came to the Laboratory, they were asked about the willingness to take part in the research study. Those who were interested were informed of the voluntary nature of their participation and their right to withdraw at any time. All participants received and signed an informed consent. Individuals who provided consent were escorted to a private area where they could sit and complete the questionnaire packet. The questionnaire packet included demographic questions (e.g., age, years of education, information about parents). Only individuals who attended school for more than 5 years and who had both parents alive until the age of 12 were selected for the final sample. The rationale behind focusing on the educational inclusion criterion was to identify a well-established level to ensure the understanding of self-rating scales. While, we selected the age of 12 as a cut-off because the most common range of the alienated child is from 8 to 14 [32, 33].

A non-random convenience sample of 554 individuals was approached for recruitment to the study. Thirty-nine individuals refused to participate citing lack of time or disinterest in the study. Therefore, 515 subjects accepted to participate and completed the demographic section of the packet to assess for the inclusion/exclusion criteria. Based on the demographic information they provided, three individuals were excluded because they only had one parent alive. A total number of 512 participants were finally recruited for this study and completed the full survey. Twenty-one cases were excluded due to incomplete data. The remaining 491 cases were used for statistical analyses. The protocol was realized according to the ethical guidelines of the Italian Association of Psychology (AIP). The study was approved by the Institutional Review Board of the Department of Psychological, Health, and Territorial Sciences, “G. d’Annunzio” University of Chieti-Pescara, Italy.

### Instruments

The survey consisted of demographic questions (i.e., age, gender, level of education, employment, and information about parents) and a series of standardized measures, three of which were examined for this study.

#### Baker Strategy Questionnaire (BSQ)

The BSQ [2] is a 20-item self-rating scale consisting of 19 items, each describing specific PA behaviors (e.g.**,** said parent was unloving; made child choose; referred to the new spouse as mom/dad) and one item referred to a general behavior that one parent tried to turn the child against the other parent (item 20). The respondents answered separately for mother and father on a five-point scale from Never (0) to Always (4). Total scores could range from 0 to 80 for each parent. The BSQ is increasingly used in other studies that have investigated PA behaviors showing a high internal consistency across samples [8, 10, 27, 28, 30]. In this study, scores across parents were combined to create a “total exposure to PA score” which could range from 0 to 160. This approach has been found to be meaningful in prior studies [29, 30] to identify the impact of PA conceived as an expression of dysfunctional parental behaviors. Moreover, studies on the effects of exposure to PA behaviors by the gender of preferred parents, have shown identical patterns [13, 29].

#### Beck Depression Inventory – II (BDI-II)

The BDI-II is a self-rating scale for measuring the severity of depression [34]. The BDI-II consists of 21 items, developed to assess symptoms corresponding to diagnostic criteria of depressive disorders listed in the Diagnostic and Statistical Manual of Mental Disorder, Fourth Edition (DSM-IV) [35]. Although the BDI-II was originally developed to measure depression symptoms according to the DSM-IV diagnostic criteria, this self-rating scale fully covers the classification of major depressive disorder as defined in the fifth edition of the DSM-5 [36].

Each item in the BDI-II is scored on a four-point Likert scale ranging from 0 to 3, with higher scores reflecting greater depressive symptomatology. Total score could range from 0 to 63. In our study, we administered the validated Italian version of the BDI-II [37]. In addition, we used the 6-item version of the BDI-II, derived from the original version in clinical validation studies [38, 39]**,** to assess core symptoms of depression. The 6-item version of the BDI-II corresponds, according to Bech et al. [14, 38], to the six core items of the Hamilton Depression Scale (HAM-D_6_) measuring the severe symptoms of depression. Six such items are the following: sadness; guilty feelings; agitation; indecisiveness; loss of energy; irritability. In the present study, the 6-item version of the BDI-II had adequate internal consistency (α = 0.77) and a high positive correlation with the original 21-item version (*r* = .92, *p* < 0.01).

#### Short-Form 36 (SF-36) health survey

The SF-36 [40, 41] is a self-report questionnaire of subjective HRQoL consisting of 36 items grouped into the following 8 domains: physical functioning (PF); role physical (RP); bodily pain (BP); general health (GH); vitality (VT); social functioning (SF); role emotional (RE); mental health (MH). PF assesses limitations in physical activities. RP measures problems with work or other daily activities as a result of physical health. BP assesses limitations due to pain. GH measures personal health and worry about changes in health. VT evaluates energy level and fatigue. SF measures the impact of physical health or emotional problems on social activities. RE evaluates problems with work or other daily activities as a result of emotional problems. MH assesses happiness, nervousness and depression. Scores on each domain range from 0 to 100. Higher scores indicate better HRQoL.

We used the Italian version of the SF-36 [42]. Further, as suggested by Bech [43], we selected three of the mental health well-being items of the WHO-5 included in the SF-36 (9d “Peaceful”, 9e “Energy”, and 9h “Happy”), for computing the score value for WHO-3. A large body of studies [43–46] showed that wording items of rating scales in opposite directions implied many psychometric disadvantages as obtaining significantly higher mean scores on negative items than on positive questions after reversing the score of these items [47]. The main reasons for focusing on three items included in the SF-36 were: (1) the mental health items of the SF-36 are positively and negatively worded covering a mixture of distress and well-being items rather than a single underlying construct of positive mental health [41]. By contrast, three items in the SF-36 (i.e., two from the MH domain and another one from the VT domain) are only positively phrased/formulated, with the total score reflecting a condition of pure psychological well-being rather than a mix of mental symptoms and quality of life aspects [44–46]. (2) Low scores on these three items (WHO-3) can be used as a screening measure [46, 48] for capturing two core symptoms of major depression (i.e., depressed mood and lack of energy) according to ICD-10 diagnostic criteria [49].

In this study, reliability of the WHO-3 was established with a Cronbach’s alpha of 0.62. Moreover, the WHO-3 showed a significant negative correlation with the BDI-II (*r* = −.53, *p* < 0.01).

### Data analysis

We scored all questionnaires both as continuous and dichotomous variables. The dichotomous BSQ score was created by establishing a value of 0 when participant reported no exposure to PA behaviors (No PA), and a value of 1 when any exposure to PA behaviors was reported (PA). This approach of transformation has been found to be meaningful in prior studies and can be useful for recognizing the impact of even the smallest doses of PA [9, 13, 30]. The transformation of BDI-II total scores was computed using established cut-off scores [43, 50] for at least moderate depression (Table 1). SF-36 domain scores were transformed using Italian normative data sample [42]. Z scores were used for establishing the presence of limitation for each HRQol domain. Finally, an established cut-off score [41] was used for the WHO-3 score to detect at least mild depression **(**Table 1**)**.

**Table 1:** Clinical cut-off scores of outcome measures

Outcomes	Cut-off scores	Meaning
BDI-II	≥ 20	At least moderate depression
BDI-6	≥ 7	At least moderate depression
WHO-3	≥ 50	At least mild depression
SF-36 PF	≤ −1^a^	Limitation in the physical functioning domain
SF-36 RP	≤ −1^a^	Limitation in the role physical domain
SF-36 BP	≤ −1^a^	Limitation in the bodily pain domain
SF-36 GH	≤ −1^a^	Limitation in the general health domain
SF-36 VT	≤ −1^a^	Limitation in the vitality domain
SF-36 SF	≤ −1^a^	Limitation in the social functioning domain
SF-36 RE	≤ −1^a^	Limitation in the role emotional domain
SF-36 MH	≤ −1^a^	Limitation in the mental health domain

^a^Z score

After transformation procedures, descriptive statistics of the sample were computed. To analyze whether depressive symptomatology and HRQoL varied by presence of PA behaviors**,** ANCOVA analyses were conducted controlling for age and parental separation/divorce. We controlled for whether the parents of the respondent had divorced/separated to test for the effects of PA over and above the effects of dissolution of the marriage. In these analyses, we used the dichotomous BSQ score as group variable and total continuous scores for the BDI-II, the 6-item version of the BDI-II, the SF-36, and the WHO-3.

To examine whether the higher exposure to PA was associated with higher scores on the outcome measures (i.e.**,** depressive symptoms and HRQoL)**,** a partial correlation was performed. In this analysis, we used the BSQ total score (i.e., total exposure to PA score), and**,** again**,** we controlled for age and whether the parents of the participant had divorced/separated.

Chi-square analyses were conducted to examine whether any exposure to PA behaviors (PA vs. No PA groups) was associated with greater likelihood of being above the clinical cut-off on the BDI-II, the 6-item version of the BDI-II, and the WHO-3, as well as with greater likelihood of being below the established threshold on SF-36 domains. For these analyses, dichotomous scores for all measures were selected.

Starting from results of chi-square analyses, binary logistic regression was used to test whether any exposure to PA behaviors (PA vs. No PA groups) was associated with mild to moderate depressive symptomatology (BDI-II, 6-item version of the BDI-II, and WHO-3 cut-off scores) as well as with low levels of HRQoL (SF-36 threshold scores for GH, VT, SF, and RE), controlling for age and whether parents were separated/divorced.

## Results

Mean age of participants was 32.43 (SD = 12.93). Most of them (59.67%) were females and had 13 years of education or less (71.69%). The 2 most frequent jobs were working (38.98%) and studying (41.22%). In 21.38% of cases, participants had parents who had separated or divorced. Demographic information for PA and No PA groups based on the BSQ dichotomous score is summarized in Table 2. The PA group had significant lower age and higher rates of parental separation/divorce than the No PA group. There were no other differences between groups on demographic variables (Table 2).

**Table 2:** Descriptive statistics for the total sample and the PA and No PA groups, and comparisons on demographic variables and outcome measures

	Total sample	PA	No PA	*F*	*d*
	*n* = 491	*n* = 319	*n* = 172		
Mean age	32.43 (12.93)	29.76 (11.89)	37.40 (13.33)	10.99**	0.60
Gender				3.46	0.17
Female	59.67	62.70	54.07		
Male	40.33	37.30	45.93		
Years of education				7.11	0.24
Five to twelve years	19.55	19.75	19.19		
Thirteen years	52.14	50.78	54.65		
More than thirteen years	28.31	29.47	26.16		
Job status				1.65	0.15
Employed	38.98	31.66	52.63		
Unemployed	19.80	18.50	22.22		
Student	41.22	49.84	25.15		
Parental separation/divorce				36.22***	0.56
No	78.62	68.97	96.51		
Yes	21.38	31.03	3.49		
BDI-II	8.61 (8.30)	9.43 (8.55)	7.10 (7.61)	5.15*	0.10
BDI-6	2.64 (2.71)	2.97 (2.78)	2.04 (2.46)	6.79**	0.14
WHO-3	52.71 (19.18)	50.58 (18.48)	56.67 (19.87)	8.67**	0.17
SF-36 PF	93.25 (10.51)	93.20 (10.78)	93.34 (10.03)	1.57	0.01
SF-36 RP	81.93 (30.27)	78.76 (31.50)	87.79 (26.97)	6.74*	0.14
SF-36 BP	78.46 (21.45)	76.62 (21.45)	81.86 (21.09)	6.34*	0.13
SF-36 GH	67.90 (17.88)	66.02 (18.33)	71.40 (16.52)	13.33***	0.27
SF-36 VT	59.71 (18.81)	57.52 (18.63)	63.75 (18.53)	6.68*	0.14
SF-36 SF	71.26 (22.76)	67.91 (22.85)	77.47 (21.32)	9.78**	0.20
SF-36 RE	67.14 (39.47)	63.11 (39.58)	74.61 (38.26)	3.39	0.01
SF-36 MH	63.16 (18.07)	60.99 (17.57)	67.19 (18.33)	9.51**	0.19

BDI-II = Beck Depression Inventory-II; BDI-6 = 6-item version of the Beck Depression Inventory-II; No PA = no exposure to Parental Alienation behaviors; PA = any exposure to Parental Alienation behaviors; SF-36 PF = Physical Functioning; SF-36 RP = Role Physical; SF-36 BP = Bodily Pain; SF-36 GH = General Health; SF-36 VT = Vitality; SF-36 SF = Social Functioning; SF-36 RE = Role Emotional; SF-36 MH = Mental Health; WHO-3 = 3-item version of the 5-item World Health Organization Well-Being Index

*p < 0.05. **p < 0.01. ***p < 0.001

Overall, ANCOVA results indicated significant group differences between PA and No PA in both BDI-II measures, WHO-3, and 6 of the 8 SF-36 domains (all but PF and RE). Mean scale scores by group are presented in Table 2.

Partial correlation analysis revealed that higher exposure to PA behaviors was significantly associated with higher scores on the BDI-II measures and lower scores on the WHO-3 (Table 3). Regarding the association with HRQoL scores, higher exposure to PA behaviors was significantly related with lower scores on 5 of the 8 SF-36 domains (RP, GH, VT, SF, and MH). Correlations were small but statistically significant.

**Table 3:** Correlations between Baker Strategy Questionnaire (BSQ) total score and outcome measures

Outcomes	Exposure to PA
BDI-II	0.10*
BDI-6	0.10*
WHO-3	−0.14**
SF-36 PF	−0.03
SF-36 RP	−0.14**
SF-36 BP	−0.08
SF-36 GH	−0.13**
SF-36 VT	−0.12**
SF-36 SF	−0.15**
SF-36 RE	−0.08
SF-36 MH	−0.12**

BDI-II = Beck Depression Inventory-II; BDI-6 = 6-item version of the Beck Depression Inventory-II; PA = total Parental Alienation score; SF-36 PF = Physical Functioning; SF-36 RP = Role Physical; SF-36 BP = Bodily Pain; SF-36 GH = General Health; SF-36 VT = Vitality; SF-36 SF = Social Functioning; SF-36 RE = Role Emotional; SF-36 MH = Mental Health; WHO-3 = 3-item version of the 5-item World Health Organization Well-Being Index

*p < 0.05. **p < 0.01

Chi-square testing showed significant differences between groups (Table 4). Specifically, the percentage of individuals that scored above the clinical cut-off for both BDI-II measures and the WHO-3 was significantly higher in the PA group than in the No PA group. Moreover, a significant higher percentage of individuals in the PA group was above the critical threshold for 4 of the SF-36 domains (GH, VT, SF, and RE) than those in the No PA group.

**Table 4:** Proportion of participants with scores above the clinical cut-off by PA and No PA groups

Outcomes			
	PA	No PA	*X* ^*2*^
BDI-II	14.11	5.81	7.67**
BDI-6	10.03	4.07	5.39*
WHO-3	50.16	38.37	6.42*
SF-36 PF	2.82	1.74	0.54
SF-36 RP	14.11	9.30	2.33
SF-36 BP	14.11	9.30	2.61
SF-36 GH	11.91	5.23	5.71*
SF-36 VT	20.69	11.05	7.18**
SF-36 SF	33.33	19.30	11.04**
SF-36 RE	36.68	26.16	5.80*
SF-36 MH	18.50	13.37	2.31

BDI-II = Beck Depression Inventory-II; BDI-6 = 6-item version of the Beck Depression Inventory-II; No PA = no exposure to Parental Alienation behaviors; PA = any exposure to Parental Alienation behaviors; SF-36 PF = Physical Functioning; SF-36 RP = Role Physical; SF-36 BP = Bodily Pain; SF-36 GH = General Health; SF-36 VT = Vitality; SF-36 SF = Social Functioning; SF-36 RE = Role Emotional; SF-36 MH = Mental Health; WHO-3 = 3-item version of the 5-item World Health Organization Well-Being Index

. *p < 0.05. **p < 0.01

Binary logistic regression analyses revealed that, controlling for all the other variables in the model (age and parental separation/divorce), there was a significant relationship between exposure to PA behaviors during childhood and increased likelihood of mild to moderate depression in adulthood (Table 5). Further, by controlling for age and parental separation/divorce, there was a significant relationship between exposure to PA behaviors and sub-optimal levels of GH and SF (Table 6).

**Table 5:** Binary logistic regression of PA, controlling for age and parental separation/divorce on mild to moderate depression

	Outcomes											
	Moderate Depression BDI-II cut-off				Moderate Depression BDI-6 cut-off				Mild Depression WHO3 cut-off			
Predictor	B	*p*	*OR*	95% CI	B	*p*	*OR*	95% CI	B	*p*	*OR*	95% CI
Age	0.01	0.40	1.01	0.99–1.04	−0.01	0.62	0.99	0.96–1.02	0.00	0.99	1.00	0.99–1.02
Parental separation/divorce	0.29	0.42	1.33	0.67–2.66	0.35	0.37	1.42	0.66–3.01	0.17	0.50	1.18	0.73–1.90
PA	0.98	0.01*	2.66	1.26-5.64	0.80	0.04*	2.23	1.12-5.41	0.44	0.04*	1.55	1.03-2.32

CI = confidence interval; *OR* = odds ratio; BDI-II = Beck Depression Inventory-II; BDI-6 = 6-item version of the Beck Depression Inventory-II; PA = total Parental Alienation score; WHO-3 = 3-item version of the 5-item World Health Organization Well-Being Index

*p < .05

**Table 6:** Binary logistic regression of PA, controlling for age and parental separation/divorce on HRQol domains

	Outcomes															
	General Health SF-36 GH threshold				Vitality SF-36 VT threshold				Social Functioning SF-36 SF threshold				Role Emotional SF-36 RE threshold			
Predictor	B	*p*	*OR*	95% CI	B	*p*	*OR*	95% CI	B	*p*	*OR*	95% CI	B	*p*	*OR*	95% CI
Age	0.01	0.70	1.01	0.98–1.03	−0.01	0.43	0.99	0.97–1.01	−0.16	0.07	0.98	0.97–1.00	−0.05	0.00**	0.96	0.94–0.97
Parental separation/divorce	0.16	0.67	1.18	0.56–2.48	0.63	0.03*	1.88	1.06–3.31	−0.11	0.69	0.90	0.54–1.50	−0.42	0.10	0.66	0.40–1.09
PA	0.89	0.03*	2.43	1.10–5.37	0.48	0.11	1.62	0.90–2.92	0.65	0.00**	1.92	1.19–3.01	0.30	0.19	1.35	0.86–2.11

*CI* confidence interval, *OR* odds ratio, *PA* total Parental Alienation score, *SF-36 GH* General Health, *SF-36 VT* Vitality, *SF-36 SF* Social Functioning, *SF-36 RE* Role Emotional

*p < .05, **p < .01

## Discussion

The current study was performed to provide further knowledge on the impact of exposure to PA behaviors on adulthood health. Results confirmed the significant association between depression and reported exposure to PA in adults [30]. Such a finding provides further evidence of this relationship corroborating it with different measures of depression. Participants who reported exposure to PA behaviors showed significantly higher levels of depressive symptoms than those who did not. The association between exposure to PA and risk of depression has been argued from a theoretical point of view and from evidence of typical patterns of psychological maltreatment [51]. Baker [1] hypothesizes that PA behaviors are likely to induce in children uncertain feelings of a parent’s love and acceptance, unnecessary and unexplained separations from an attachment figure (the targeted parent), and cognitive dissonance experiences. Moreover, the relationship with the preferred parent can cause the emergence of memories of being unloved, rejected, threatened, criticized, subordinate, and alone. These feelings, memories, and experiences can result in internalized models of uncertainty about themselves and the world as well as in various submissive and defensive behaviors. A large body of literature established that cognitive biases that seem to be supported by negative self-schemata [52] and submissive behaviors are highly associated with depression [53]. However, these hypotheses were not tested in this study. Since the association between exposure to PA and depression is now well-established, future studies should identify potential mediators or moderators of this relationship.

To further uncover long-term outcomes of perceived PA behaviors during childhood, the relationship between exposure to PA and HRQoL domains was examined. We chose to adopt the SF-36 as a well-established measure developed in the International Quality of Life Assessment (IQOLA) project [54] since HRQoL is a topic in which cultural beliefs, behaviors and bias may affect the responses of participants, raising the issue of cross-cultural equivalence of translated measures [55, 56]. The IQOLA project was launched with the goal of testing the cross-cultural applicability of the SF-36 with a team of investigators from 14 different countries. The findings from several studies demonstrated that the Italian version of the SF-36 had a high level of equivalence with the original version developed in the United States [42].

The findings of the present study showed that reported exposure to PA was associated with lower HRQoL. Our data highlighted that the burden was mostly associated with psychological health domains, but also appeared for general health. Specifically, perceiving an exposure to PA increased the odds of diminished social functioning (SF) and general health (GH). High scores on these domains indicated that individuals exposed to PA behaviors were more likely to believe personal health was poor and can get worse, and to experience extreme and frequent interference with social activities because of physical and emotional problems.

A possible explanation for these findings is that children who perceived PA - conceived as a form of PM - may learn maladaptive ways of coping with life experiences. This, in turn, could affect the way they see themselves and others, with potential negative consequences on their daily life. Previous studies highlighted that psychologically maltreated individuals may use maladaptive coping strategies compared to those with lower levels of psychological maltreatment [57]. Moreover, maladaptive coping strategies are related to poor mental health and well-being against stressful life experiences [58, 59]. According to Verrocchio et al. [12] individuals exposed to PA behaviors have a higher likelihood of developing low self-esteem, perceiving only negative aspects of situations or having a poor perception of one’s capacity to cope with stressing environments. Oriented by previous studies on outcomes of psychological maltreatment [22], our results add to the growing body of knowledge about the impact of exposure to PA on adult well-being.

The study is not without methodological limitations which must be considered when interpreting the results. The use of a nonrandom sampling method limits the generalizability of the findings outside of this sample. Therefore, the findings need to be validated in a well-controlled clinical study. Even though we assessed all key variables using reliable and validated instruments, the findings may be subjected to recall bias. However, previous studies have shown that the size of any recall bias was small when assessing association between childhood maltreatment and health-related outcomes [60, 61]. In addition, the cross-sectional design did not permit us to make conclusions about causal effects since the exposure and outcome variables were collected simultaneously. These limitations notwithstanding, our study makes a novel contribution to the literature addressing long-term health outcomes of PA. It is indeed the first study to assess the relationship between perceived exposure to PA behaviors during childhood and HRQoL in adulthood. Future research efforts could continue to explore the negative outcomes of PA on children’s well-being with data from hetero-evaluation measures or by a prospective observational design.

## Conclusions

Childhood exposure to PA behaviors was related to higher likelihood of depressive symptoms and diminished HRQoL in adulthood. Children need to be identified as victims of PA to protect them from future negative outcomes. Further research efforts should be focused on short- and long-term studies to demonstrate the magnitude of PA in at-risk children and subsequent interventions to reduce additional exposure as well as interventions to minimize the long-term impact for at-risk children.
